# Commonly Used Dietary Supplements on Coagulation Function during Surgery

**DOI:** 10.3390/medicines2030157

**Published:** 2015-07-27

**Authors:** Chong-Zhi Wang, Jonathan Moss, Chun-Su Yuan

**Affiliations:** Department of Anesthesia & Critical Care, University of Chicago, Chicago, IL 60637, USA; E-Mails: JM47@dacc.uchicago.edu (J.M.); CYuan@dacc.uchicago.edu (C.-S.Y.)

**Keywords:** dietary supplements, natural products, surgery, coagulation, platelet function, bleeding

## Abstract

**Abstract:**

**Background:**

Patients who undergo surgery appear to use dietary supplements significantly more frequently than the general population. Because they contain pharmacologically active compounds, dietary supplements may affect coagulation and platelet function during the perioperative period through direct effects, pharmacodynamic interactions, and pharmacokinetic interactions. However, in this regard, limited studies have been conducted that address the pharmacological interactions of dietary supplements. To avoid possible bleeding risks during surgery, information about the potential complications of dietary supplements during perioperative management is important for physicians.

**Methods:**

Through a systematic database search of all available years, articles were identified in this review if they included dietary supplements and coagulation/platelet function, while special attention was paid to studies published after 1990.

**Results:**

Safety concerns are reported in commercially available dietary supplements. Effects of the most commonly used natural products on blood coagulation and platelet function are systematically reviewed, including 11 herbal medicines (echinacea, ephedra, garlic, ginger, ginkgo, ginseng, green tea, kava, saw palmetto, St John’s wort, and valerian) and four other dietary supplements (coenzyme Q_10_, glucosamine and chondroitin sulfate, fish oil, and vitamins). Bleeding risks of garlic, ginkgo, ginseng, green tea, saw palmetto, St John’s wort, and fish oil are reported. Cardiovascular instability was observed with ephedra, ginseng, and kava. Pharmacodynamic and pharmacokinetic interactions between dietary supplements and drugs used in the perioperative period are discussed.

**Conclusions:**

To prevent potential problems associated with the use of dietary supplements, physicians should be familiar with the perioperative effects of commonly used dietary supplements. Since the effects of dietary supplements on coagulation and platelet function are difficult to predict, it is prudent to advise their discontinuation before surgery.

## 1. Introduction

Complementary and alternative medicine (CAM) comprises health care practices that are not currently considered an integral part of conventional therapies [1,2,3]. CAM covers a wide spectrum of ancient to modern approaches that purport to expand options for preventing and treating diseases [4,5]. Some people use CAM to relieve the side effects of conventional treatment or to enhance its effects [6,7]. Others use CAM as a means to increase their sense of control over health care [8,9]. 

While CAM offers patients more health care options, it is not without risks [10,11]. Not only can CAM result in direct harm, but also the interactions between CAM and conventional medications may delay necessary medical therapies [12,13]. Because knowledge of CAM is limited, in many cases, the benefits and risks remain only partially understood. Studies have also confirmed that physicians and other health care professionals have a poor knowledge of CAM [14,15].

Dietary supplements, including herbal medicine derived natural products, are a very commonly used modality in CAM [16,17] and are often used by those who undergo surgical procedures. For example, surgical patients appear to use herbal medicines significantly more frequently than the general population. Tsen et al. reported that 22% of patients who underwent evaluation in a preoperative clinic took herbs [18]. Kaye *et al.* found that 32% of patients in an ambulatory surgery setting admitted to using herbal medications [19]. In a recent retrospective review, 23% of surgical patients indicated the use of natural products, and older patients preferred dietary supplements [20].

Although the FDA has released Current Good Manufacturing Practices (CGMPs) for Dietary Supplements [21], product variability exists between manufacturers. Because dietary supplements contain pharmacologically active agents, they may affect blood coagulation and platelet function [22,23,24] through several mechanisms, including direct effects (e.g., intrinsic pharmacologic effects), pharmacodynamic interactions (e.g., alteration of the action of conventional drugs at effector sites), and pharmacokinetic interactions (e.g., alteration of the absorption, distribution, metabolism, elimination, and toxicity of conventional drugs). A relatively small number of studies have successfully addressed the pharmacological interactions of herbal remedies [25,26]. Although limited, a pharmacokinetic analysis of the different constituents in herbal remedies would be useful to clinicians [27,28].

Recommendations for the use of dietary supplements are often based on small clinical trials, case reports, animal studies, or predictions derived from known pharmacology and expert opinions. Research is essential because natural products are often widely adopted by the public before adequate data support their safety and efficacy [29,30]. A mini review discussed the effects of herbal remedies on the coagulation cascade, however, most information in the article relied on *in vitro* evidence [31].

In this article, we do not present a comprehensive review of dietary supplements. Rather, we consider specific components relevant to the effects of natural products on hemodynamics, coagulation and platelet function, focusing primarily on the most commonly used herbal medicines and other dietary supplements based on two comprehensive CAM surveys in the United States.

## 2. Methods

### 2.1. Database and Search Strategies

A systematic search was performed in PubMed, SciFinder and Web of Science (1970–2014), with the following terms: (natural product or herbal medicine or dietary supplement or selected natural products) and (coagulation or platelet function or perioperative care). All of those searches ended before December 2014, while special attention was paid to studies published after 1990. In addition, we also performed manual searches in related textbooks and recent surgery and alternative medicine journals. If necessary, further searches were conducted based on information in important references.

### 2.2. Fundamental CAM Information from Two Surveys in the U.S.

To evaluate whether CAM has become an integral part of American health care, the National Institutes of Health conducted two comprehensive surveys in 2002 and 2007. In the 2002 survey, 36.0% of 31,044 adults interviewed had used some form of CAM in the previous 12 months [32]. In the 2007 survey, 38.3% of 23,393 adults interviewed had used CAM in the previous year [33]. In the 2007 survey, the most commonly used CAM included natural products (17.7%), deep breathing exercises (12.7%), meditation (9.4%), chiropractic or osteopathic manipulation (8.6%), massage (8.3%), and yoga (6.1%). Between 2002 and 2007, the use of acupuncture, deep breathing exercises, massage therapy, meditation, and yoga increased [33].

### 2.3. Selection of Monographs of Natural Products in this Review

The most commonly used CAM modalities are natural products, e.g., herbal medicines and other dietary supplements [33]. The top ten herbal medicines are echinacea, ginseng, combination herb pills, ginkgo, garlic, green tea, saw palmetto, soy isoflavones, grape seed extract, and milk thistle. The top ten dietary supplements include fish oil or omega 3 or DHA, glucosamine, flaxseed oil, chondroitin, coenzyme Q_10_, fiber or psyllium, cranberry, melatonin, methylsulfonylmethane (MSM), and lutein [33]. After considering the products that have shown the greatest impact on perioperative patient care, eleven herbal medicines (echinacea, ephedra, garlic, ginger, ginkgo, ginseng, green tea, kava, saw palmetto, St John’s wort, and valerian) and four dietary supplements (coenzyme Q_10_, glucosamine and chondroitin sulfate, fish oil, and vitamins) were selected based on the data from the 2007 Survey and the latest statistical commercial information [34]. 

## 3. Results

### 3.1. Preoperative Management for Using of Dietary Supplements

Although a preoperative assessment should address the patient’s use of dietary supplements, a 2005 study showed that 90% of anesthesiologists do not routinely ask about herbal medicine usage, and 82% felt their knowledge of herbal medicines and their implications in patient care were inadequate [35]. Moreover, more than 70% of patients are not forthcoming about their herbal medicine use during routine preoperative assessment [19]. In addition to prescription and over the counter medicines, patients should be asked to bring their herbal medicines and other dietary supplements with them at the time of the preoperative evaluation [36]. A positive history of herbal medicine use should also prompt anesthesiologists to suspect the presence of undiagnosed disorders causing symptoms that lead to self-medication. Patients who use herbal medicines may be more likely than those who do not to avoid conventional diagnosis and therapy [37].

In general, the use of herbal medicines should be discontinued preoperatively. When pharmacokinetic data for the active constituents in an herbal medication are available, the timeframe for preoperative discontinuation can be tailored. For other herbal medicines, two weeks is recommended [38]. However, in clinical practice, because many patients require nonelective surgery, are not evaluated until the day of surgery, or are noncompliant with instructions to discontinue herbal medications preoperatively, they may take herbal medicines until the day of surgery. In this situation, anesthesia can usually proceed safely at the discretion of the anesthesiologist, who should be familiar with commonly used herbal medicines to avoid or recognize and treat complications that may arise [39,40]. For instance, recent use of herbal medicines that inhibit platelet function (e.g., garlic, ginseng, or ginkgo) may warrant specific strategies for procedures with substantial intraoperative blood loss (e.g., platelet transfusion) and those that alter the risk–benefit ratio of using certain anesthetic techniques (e.g., neuraxial blockade) [25].

Preoperative discontinuation of all herbal medicines may not eliminate complications associated with their use. Withdrawal of regular medications is associated with increased morbidity and mortality after surgery. In general, medications with the potential to induce rebound and withdrawal symptoms should be continued. The use of noncritical medications that can increase surgical risk should be discontinued. If neither of these conditions apply, consider the patient’s risk profile and the risk of the procedure when making perioperative management decisions [41]. In most cases, herbal medicines should be discontinued before surgery because they are not critical. Abstinence after long-term use of an herbal medicine such as valerian has the potential to produce acute withdrawal.

### 3.2. Commonly Used Herbal Medicines

#### 3.2.1. Echinacea

*Echinacea* is a genus of herbaceous flowering plants in the family *Asteraceae*. Its nine species are commonly called purple coneflowers. Three species, *Echinacea angustifolia*, *Echinacea purpurea*, and *Echinacea pallida*, are used for prophylaxis and treatment of viral, bacterial, and fungal infections, particularly those of upper respiratory origin, although its efficacy in the latter is doubtful [42]. A 2008 meta-analysis showed echinacea’s benefit in decreasing the incidence and duration of the common cold [43]. Pharmacological activity cannot be attributed to a single compound, although the lipophilic fraction, which contains alkylamides, polyacetylenes and essential oils, appears to be more active than the hydrophilic fraction [44].

Echinacea had a number of immunostimulatory effects in early preclinical studies [45]. While there are no studies specifically addressing interactions between echinacea and immunosuppressive drugs, expert opinion generally warns against the concomitant use of echinacea and these drugs, owing to the probability of diminished effectiveness [46]. In contrast to the immunostimulatory effects with short-term use, later observations suggested that long-term use of more than eight weeks has the potential for immunosuppression [46] and a theoretically increased risk of certain complications such as poor wound healing and opportunistic infections. A recent phytochemical study identified a potential immunosuppressant compound cynarin from echinacea [47]. The biological activity of echinacea could be immunostimulatory, immunosuppressive, or anti-inflammatory depending on the portion of the plant and extraction method [48].

Echinacea has also been associated with allergic reactions, including one reported case of anaphylaxis [49]. Therefore, echinacea should be used with caution in patients with asthma, atopy, or allergic rhinitis. Concerns of potential hepatotoxicity have also been raised, although documented cases are lacking [50]. In the absence of definitive information, patients with pre-existing liver dysfunction should be cautioned against using echinacea. Although several *in vitro* and *in vivo* pharmacokinetic studies on echinacea have been reported, information about the pharmacokinetics of echinacea is still limited [51]. Echinacea significantly reduced plasma concentrations of S-warfarin, but did not significantly affect warfarin pharmacodynamics and platelet aggregation in healthy subjects [52]. 

#### 3.2.2. Ephedra

Ephedra, known as *ma huang* in Chinese medicine, comprises the aerial parts of three principal plant species: *Ephedra sinica*, *Ephedra equisetina*, and *Ephedra intermedia var. tibetica*. Ephedra contains alkaloids including ephedrine, pseudoephedrine, norephedrine, methylephedrine, and norpseudoephedrine [53]. In Chinese medicine, it is used to treat respiratory conditions, such as asthma and bronchitis. Ephedra-containing dietary supplements were once widely used in the U.S. to promote weight reduction and enhance energy. In one clinical study, the combination of caffeine and ephedra significantly increased resting energy expenditure, heart rate and blood pressure [54]. Publicity about adverse reactions to this herb prompted the FDA to ban its sale in 2004, but ephedra is still widely available via the Internet.

Ephedra causes dose-dependent increases in blood pressure and heart rate. Ephedrine, the predominant active compound, is a non-catecholamine sympathomimetic that exhibits α_1_, β_1_ and β_2_ activity directly at adrenergic receptors and indirectly by releasing endogenous norepinephrine (noradrenaline). These sympathomimetic effects have been associated with more than 800 reported adverse events, including fatal cardiac and central nervous system complications [55].

Ephedra may affect cardiovascular function and vasoconstriction, and, in some cases, vasospasm of coronary and cerebral arteries may cause myocardial infarction and thrombotic stroke [56]. Ephedra also caused hypersensitivity myocarditis, characterized by cardiomyopathy with myocardial lymphocyte and eosinophil infiltration [25]. Concomitant use of ephedra and monoamine oxidase inhibitors can result in life-threatening hyperpyrexia, hypertension, and coma [57]. 

In humans, ephedrine has an elimination half-life of 5.2 h with 70%–80% of the excreted compound remaining unchanged in the urine [58]. Based upon the pharmacokinetic data and the known cardiovascular risks of ephedra, including myocardial infarction, stroke and cardiovascular collapse from catecholamine depletion [57], this herb should be not used as a dietary supplement.

#### 3.2.3. Garlic

Garlic (*Allium sativum*) has the potential to modify the risk of developing atherosclerosis by reducing blood pressure, thrombus formation, and serum lipid and cholesterol levels [59]. These effects are primarily attributed to the sulfur-containing compounds, particularly allicin and its transformation products. Commercial garlic preparations may be standardized to a fixed alliin and allicin content [60].

Garlic inhibits platelet aggregation *in vivo* in a dose-dependent fashion [61,62]. The effect of one of its constituents, ajoene, appears to be irreversible and may potentiate the effect of other platelet inhibitors such as prostacyclin, forskolin, indomethacin, and dipyridamole [63]. Although these effects have not been consistently demonstrated in clinical trials [64], there are several cases in the literature on excessive dietary garlic intake or use of garlic as a medicine associated with coagulation alterations [65]. One case report showed an interaction between garlic and warfarin, resulting in an increased INR [66]. In addition to bleeding concerns, garlic has the potential to decrease systemic and pulmonary vascular resistance in laboratory animals, an effect that was observed in clinical studies as well [67].

In an early study in rats, alliin was absorbed quickly after oral administration and eliminated after 6 h. Allicin was absorbed slowly after oral administration, and its plasma peak level appeared between 0.5 h and 2 h. Even four days later, allicin could still be detected in the rats [68]. Although in one clinical study garlic oil selectively inhibited CYP2E1 activity [69], it is still difficult to predict drug interactions with garlic [70,71].

#### 3.2.4. Ginger

Ginger (*Zingiber officinale*), a popular spice, has a long history of use in Chinese, Indian, Arabic, and Greco-Roman herbal medicines. Ginger has a wide range of reported health benefits for those with arthritis, rheumatism, sprains, muscular aches, pains, sore throats, cramps, constipation, indigestion, nausea, vomiting, hypertension, dementia, fever, infectious diseases, and helminthiasis [72,73,74]. Ginger contains up to 3% volatile oil, mostly monoterpenoids and sesquiterpenoids [75]. Gingerols are representative compounds in ginger [76].

Ginger is an anti-emetic and has been used to treat motion sickness and to prevent nausea after laparoscopy [77]. In an *in vitro* study, gingerols and related analogues inhibited arachidonic acid-induced human platelet serotonin release and aggregation, with a potency similar to that of aspirin [76]. In another *in vitro* study, the antiplatelet effects of 20 ginger constituents were evaluated. Five constituents showed antiplatelet activities at relatively low concentrations. One of the ginger compounds (8-paradol) was the most potent COX-1 inhibitor and antiplatelet aggregation agent [78]. In a case report, a ginger-phenprocoumon combination resulted in an elevated INR and epistaxis [79]. Although the sample size was relatively small, the platelet inhibition potential of ginger has been supported in a pilot clinical study [80]. 

#### 3.2.5. Ginkgo

Ginkgo is derived from the leaf of *Ginkgo biloba*, the sole surviving species of the large plant division *Ginkgophyta*, once widespread throughout the world 180 million years ago. Ginkgo extracts used as medicine contain terpene trilactones, flavonoids, biflavones, proanthocyanidins, alkylphenols, and polyprenols [81,82]. Ginkgo has been used for cognitive disorders, peripheral vascular disease, age-related macular degeneration, vertigo, tinnitus, erectile dysfunction, and altitude sickness [83,84,85]. Studies have suggested that ginkgo may stabilize or improve cognitive performance in patients with Alzheimer’s disease and multi-infarct dementia [86] but not in healthy geriatric patients [87]. When plasma and brain levels of terpene trilactones were evaluated *in vivo*, ginkgolides A and B and bilobalide crossed the blood-brain barrier after a single oral dose of ginkgo extract [88]. Because the compounds believed to be responsible for its pharmacological effects are the terpene trilactones and flavonoids, most commercial ginkgo extracts are standardized for their content of these compounds [81].

Ginkgo appears to alter vasoregulation, act as an antioxidant, modulate neurotransmitter and receptor activity and inhibit platelet-activating factor (PAF). Of these effects, the inhibition of PAF raises the greatest concern for surgical patients [38]. Ginkgo showed antiplatelet and antithrombotic effects in *in vivo* animal models [89,90]. In a clinical trial, ginkgo reduced platelet aggregation but had no effect on coagulation assays or bleeding time [91]. In a study of 50 healthy male volunteers, 29 received 2 × 120 mg/day of ginkgo extract for seven days; however, none in the ginkgo group had abnormal platelet and coagulation parameters [92]. Despite lack of evidence from a clinical trial, ten case reports have implicated ginkgo as a cause of catastrophic bleeding [93,94], including one case of spontaneous hyphema [95], one case of postoperative bleeding after laparoscopic cholecystectomy [96], and one of cerebral bleeding without structural abnormalities in the patient [94].

Terpene trilactones are highly bioavailable when administered orally. The elimination half-lives of the terpene trilactones after oral administration are between 3 h and 10 h. For ginkgolide B, a dosage of 40 mg twice daily resulted in a higher area under the curve, and a longer half-life and residence time than after a single 80 mg dose. A once daily dose of 80 mg guaranteed a higher maximum concentration peak (T_max_). T_max_ was reached 2–3 h after administration with both dosages [97].

#### 3.2.6. Ginseng

Among the several species of ginseng used for their pharmacological effects, Asian ginseng (*Panax ginseng*) and American ginseng (*Panax quinquefolius*) are the most commonly described [17,98]. Ginseng has been labeled an “adaptogen” since it reportedly protects the body against stress and restores homeostasis [99,100,101]. Because its pharmacological actions are attributed to the ginsenosides, a group of steroidal saponin compounds, many commercially available ginseng preparations have been standardized to ginsenoside content [17,102].

The many heterogeneous and sometimes opposing effects of different ginsenosides [103,104] give ginseng a broad but incompletely understood pharmacological profile with regards to its effects on general health, fatigue, immune function, cancer, cardiovascular disease, diabetes mellitus, cognitive function, viral infections, sexual function, and athletic performance [99]. The underlying mechanism appears to be similar to that classically described for steroid hormones. A potential therapeutic use for this herb lies in its ability to lower postprandial blood glucose in both type 2 diabetics and non-diabetics [105,106], an effect that may create unintended hypoglycemia in patients who have fasted before surgery. In a recent case report, after the oral administration a large amount of ginseng, a female patient with no cardiovascular risk factors developed prolonged QT with subsequent Torsades de pointes, suggesting that a high dose of ginseng may have a risk of inducing arrhythmia [107].

There is concern about ginseng’s effect on hemostasis. One early study suggested that the antiplatelet activity of panaxynol, a constituent of ginseng, may be irreversible in humans [108]. Other studies found that ginseng extract and ginsenosides inhibit platelet aggregation *in vitro* [109,110], and prolong both thrombin time and activated partial thromboplastin time in *in vivo* animal models [111,112]. The clinical evidence implicating ginseng as a cause of bleeding is weak and based on only a few case reports [93]. Although ginseng may inhibit the coagulation cascade, in one case its use was associated with a significant decrease in warfarin anticoagulation [113]. Subsequently, a study involving volunteers showed that American ginseng interfered with warfarin-induced anticoagulation [114]. In another clinical trial, warfarin’s apparent clearance was moderately increased with Asian ginseng [115]. Because warfarin is often used after orthopedic or vascular procedures, this drug interaction may affect the outcome of the procedure.

In rats, after an intravenous infusion of ginseng, ginsenosides Re and Rg1 were eliminated quickly from the body with a T_1/2_ between 0.7 h and 4 h; ginsenosides Rb1 and Rd were eliminated slowly from the body with a T_1/2_ between 19 h and 22 h [116]. After the oral administration of ginseng, ginsenoside Rb1 reached the maximum plasma concentration at approximately 4 h with a prolonged half-life [117,118].

#### 3.2.7. Green Tea

Tea from *Camellia sinensis* is one of the most ancient drinks and the second most widely consumed beverage in the world [119,120]. Tea can be classified into three types: green, oolong, and black. Green tea, which is non-fermented and derived directly from drying and steaming fresh tea leaves, contains polyphenolic compounds. The catechins in green tea account for 16%–30% of its dry weight. Epigallocatechin-3-gallate (EGCG), the most predominant catechin in green tea, is responsible for much of the biological activity mediated by green tea [120].

In an early *in vitro* and *in vivo* study, both green tea and EGCG significantly prolonged mouse tail bleeding time in conscious mice. They inhibited adenosine diphosphate- and collagen-induced rat platelet aggregation in a dose-dependent manner [121]. The antiplatelet activity may result from the inhibition of thromboxane A2 formation. Because ATP release from a dense granule is inhibited by catechins in washed platelets, thromboxane A2 formation may have been inhibited by preventing arachidonic acid liberation and thromboxane A2 synthase [122,123]. Regarding a possible adverse effect of green tea on platelets, one case report showed that after a patient consumed a weight-loss product containing green tea, thrombotic thrombocytopenic purpura developed [124]. Since green tea contains vitamin K, drinking green tea may antagonize the anticoagulant effects of warfarin [125].

In a randomized, double-blind, placebo-controlled study, eight subjects received oral EGCG in a single dose of 50–1600 mg. In each dosage group, the kinetic profile revealed rapid absorption with a one-peak plasma concentration *versus* time course, followed by a multiphasic decrease consisting of a distribution phase and an elimination phase. The mean half-life values observed were between 1.9 h and 4.6 h [126]. In another pilot clinical study, after five healthy subjects took tea extract orally, the concentration of EGCG in plasma was determined; the half-life of EGCG was between 2.2 h and 3.4 h [127].

#### 3.2.8. Kava

Kava is derived from the dried root of the pepper plant *Piper methysticum*. Kava has gained popularity as an anxiolytic and sedative, and kavalactones appear to be the source of kava’s pharmacological activity [53].

Kava possesses psychomotor activities. The kavalactones have dose-dependent effects on the central nervous system including antiepileptic, neuroprotective, and local anesthetic properties [128,129]. Kava may act as a sedative or hypnotic by potentiating inhibitory neurotransmission of γ-aminobutyric acid (GABA) [130]. The kavalactones increased barbiturate sleep time in laboratory animals [131]. This effect may explain the mechanism underlying the report of a coma attributed to an alprazolam–kava interaction [132]. Although kava has abuse potential, whether long-term use can result in addiction, tolerance, and acute withdrawal after abstinence has not been satisfactorily investigated. Continuous kava use may elevate gamma glutamyl transpeptidase (GGT) levels, raising concerns about hepatotoxicity [133]. In addition, the hepatotoxicity of kava may be associated with the inappropriate use of plant products and processes [134]. With continuous use, kava produces “kava dermopathy”, characterized by reversible scaly cutaneous eruptions [135].

In an *in vitro* investigation, a kava compound (+)-kavain suppressed the aggregation of human platelets [136]. A pilot survey of extensive usage of kava showed that kava users were more likely to complain of poor health and had increased red-cell volume and decreased platelet volume [137]. Kava inhibits cyclooxygenase to potentially decrease renal blood flow and interfere with platelet aggregation. Consumption of kava has potential cardiovascular effects that could manifest and the elimination half-life of kavalactones is 9 h [138]. The kavalactone compound kawain was well absorbed after oral administration during surgery [139]. In spite of safety concerns, including previous reports of liver toxicity associated with kava products in humans [140,141], kava is still available in the United States.

Peak plasma levels occur 1.8 h after an oral dose, and >90% of the dose and its metabolites were eliminated within 72 h [142]. 

#### 3.2.9. Saw Palmetto

Saw palmetto (*Serenoa repens*) is a dwarf palm tree native to Florida and other parts of the southeastern USA. The dried ripe berry extract of saw palmetto is used by men in the United States to treat symptoms associated with benign prostatic hypertrophy (BPH) [143]. The major constituents of saw palmetto are fatty acids and their glycerides (*i.e.*, triacylglycerides and monoacylglycerides), carbohydrates, steroids, flavonoids, resin, pigment, tannin, and volatile oil. The pharmacological activity of saw palmetto has not been attributed to a single compound [53].

The mechanisms of saw palmetto, including antiandrogenic, anti-inflammatory, and antiproliferative effects mediated through the inhibition of growth factors, have been studied *in vitro* [144]. An *in vivo* study revealed that at a clinically relevant dose, saw palmetto exerts a direct effect on the pharmacological receptors in the lower urinary tract, thereby improving urinary dysfunction in patients with BPH and overactive bladders [145]. The potential of saw palmetto for relieving BPH symptoms could be achieved by multiple synergistic mechanisms.

Platelet function was not affected by the administration of saw palmetto in a clinical study [93], and two case reports provided little evidence to implicate saw palmetto as a cause of bleeding. In a patient undergoing a craniotomy, however, saw palmetto was associated with excessive surgical bleeding that required termination of the procedure. This complication was attributed to saw palmetto’s anti-inflammatory effects, specifically the inhibition of cyclooxygenase and subsequent platelet dysfunction [146]. Another case of hematuria and coagulopathy in a patient who used saw palmetto was reported [147]. 

#### 3.2.10. St John’s Wort

St John’s wort is the common name for *Hypericum perforatum*, primarily used as an antidepressant. The popularity of St John’s wort is reflected in the large number of clinical trials, meta-analyses and reviews of its effectiveness as an antidepressant. The herb is more effective than a placebo in the treatment of mild to moderate depression and the incidence of side effects is low. In the treatment of major depression, however, St John’s wort is less effective [148]. The compounds believed to be responsible for pharmacological activity are hypericin and hyperforin [149]; thus, commercial preparations are often standardized for the content of the two compounds [150].

St John’s wort inhibits serotonin, norepinephrine and dopamine reuptake [151]. Concomitant use of this herb with or without serotonin reuptake inhibitors may create a syndrome of central serotonin excess [152]. Although *in vitro* data implicated monoamine oxidase inhibition as a possible mechanism of action, a number of later investigations have demonstrated that monoamine oxidase inhibition with St John’s wort is insignificant *in vivo* [153].

The use of St John’s wort can significantly increase the metabolism of many other drugs [70,154]. For example, it induces the cytochrome P450 3A4 isoform, approximately doubling its metabolic activity [155,156]. Interactions with substrates of the 3A4 isoform including indinavir sulfate [157], ethinylestradiol [158], and cyclosporine have been documented. In two case reports of heart transplant patients, after taking St John’s wort, their plasma cyclosporine concentrations became subtherapeutic and acute transplant rejection was shown. After stopping St John’s wort, plasma cyclosporine remained within the therapeutic range with no further episodes of rejection [159]. In one series of 45 organ transplant patients, St John’s wort was associated with an average decrease of 49% of cyclosporine levels in blood [160]. Another group reported two cases of acute heart transplant rejection associated with this particular pharmacokinetic interaction [159]. Other P450 3A4 substrates are alfentanil, midazolam, lidocaine, calcium channel blockers and 5-hydroxytryptamine receptor antagonists. In addition to the 3A4 isoform, the cytochrome P450 2C9 isoform also may be induced. The anticoagulant effect of warfarin, a substrate of the 2C9 isoform, was reduced in seven reported cases [158]. Other 2C9 substrates include the non-steroidal anti-inflammatory drugs. Furthermore, enzyme induction by St John’s wort may be more pronounced when other herbal enzyme inducers are taken concomitantly. St John’s wort also affects digoxin pharmacokinetics [161]. In rats, St John’s wort markedly altered hepatocyte intracellular accumulation of irinotecan and its major metabolite (SN-38) and the glucuronidation of SN-38 [162]. 

Two clinical trials showed that St John’s wort increased estrogen metabolism and caused breakthrough menstrual bleeding in users of estrogen-based oral contraceptives [163,164]. In one case report, St John’s wort may have caused severe unilateral epistaxis requiring surgical arrest of bleeding via endoscopic sphenopalatine artery ligation and anterior ethmoidal artery ligation [165].

Several clinical evaluations of the single-dose and steady-state pharmacokinetics of hypericin and hyperforin have been conducted in humans [166]. After oral administration, peak plasma levels of hypericin and hyperforin were obtained between 2.5–8.1 h and 2.5–4.4 h, respectively, and their median elimination half-lives were 6.0–48.2 h and 8.5–19.6 h, respectively. 

#### 3.2.11. Valerian

Valerian, which consists of the root of *Valeriana officinalis* and a number of other *Valeriana* species, is an herb native to the temperate areas of the Americas, Europe, and Asia [53]. It is used as a sedative, particularly in the treatment of insomnia, and virtually all herbal sleep-aids contain valerian [167]. The compounds in valerian act synergistically, but the sesquiterpenes are the primary source of valerian’s pharmacological effects. Commercially available preparations may be standardized to valerenic acid [168].

Valerian produces dose-dependent sedation and hypnosis [169]. These effects appear to be mediated through the modulation of GABA neurotransmission and receptor function [170]. Valerian increased barbiturate sleep time in experimental animals [171]. In several randomized, placebo-controlled trials in humans, there is a mild subjective improvement in sleep with valerian, especially when used for two weeks or more [172,173]. Objective tests have had less consistent results with little or no improvement in sleep noted [174]. In one patient, valerian withdrawal appeared to mimic an acute benzodiazepine withdrawal syndrome characterized by delirium and cardiac complications after surgery. Symptoms were attenuated by benzodiazepine administration [175]. Based upon these findings, valerian is expected to potentiate the sedative effects, like midazolam, via the GABA receptor.

The pharmacokinetics of valerian were evaluated in two clinical studies. In five healthy adults, serum concentration of valerenic acid was at maximum between 1 and 2 h after administration. Valerenic acid was measurable in serum for at least 5 h after the valerian dose [176]. In another study, plasma peak levels appeared between 0.47 h and 1.7 h. The large variability in the pharmacokinetics of valerenic acid may contribute to the inconsistencies in its effect as a sleep aid [177].

Most of the published clinical trials do not record side effects of valerian. In a trial involving 61 patients, there were only three cases of side-effects: two of headache and one of morning hang-over [169]. Another possible rare adverse effect of valerian is hepatotoxicity, found in one case report [178]. Abrupt discontinuation in patients who may be physically dependent on valerian risk benzodiazepine-like withdrawal. Based upon the mechanism of action and a reported case of efficacy [175], benzodiazepines can be used to treat valerian withdrawal symptoms.

#### 3.2.12. Other Herbal Medicines

In the survey conducted in 2007, the top ten herbal medicines also included soy isoflavones, grape seed extract, and milk thistle. There is no report on adverse effects related to coagulation and platelet function for these herbs. Antiplatelet aggregation activity and herb–drug interactions, however, were found in other herbs including boldo (*Peumus boldus*), Danshen (*Salvia miltiorrhiza*), Dong quai (*Angelica sinensis*) and papaya (*Carica papaya*) [179].

Danshen is a very commonly used Chinese herbal medicine for cardiovascular conditions [180,181,182]. In a clinical trial, this herbal medicine has been shown to be effective in reducing skin flap ischemia and necrosis after mastectomy [183]. The effects of antiplatelet aggregation of Danshen have been reported from a number of investigations. An *in vitro* study demonstrated that the intracellular signaling pathway involved in the antiplatelet action of tanshinone IIA could modulate tubulin acetylation and inhibit Erk-2 phosphorylation [184,185]. Cryptotanshinone is another pharmacologically active compound identified from Danshen. In a very recent study, Maione *et al.* reported that cryptotanshinone inhibited rat platelet aggregation and endowed of Gi-coupled P2Y12 receptor antagonist [186].

### 3.3. Other Commonly Used Dietary Supplements

#### 3.3.1. Coenzyme Q_10_

Coenzyme Q_10_ (CoQ_10_), or ubidecarenone, is a single-constituent antioxidant compound structurally related to vitamin K. Endogenous CoQ_10_ may prevent the membrane transition pore from opening, as it counteracts several apoptotic events, such as DNA fragmentation, cytochrome c release, and membrane potential depolarization [53].

The interaction between CoQ_10_ and warfarin was investigated in rats [187]. Following the oral administration of 1.5 mg/kg of racemic warfarin to rats during an eight-day oral regimen of CoQ_10_ (10 mg/kg daily), no apparent effect was observed on the serum protein binding of warfarin enantiomers. Treatment with CoQ_10_ did not affect the absorption and distribution of the *S*- and *R*-enantiomers of warfarin but increased total serum clearance of both *R*- and S-warfarin. The increased clearance values could be attributed to the acceleration of certain metabolic pathways and renal excretion of the warfarin enantiomers.

An *in vitro* study using human liver microsomes led to a relatively accurate pharmacokinetic prediction of CoQ_10_ activity. A 32% and 17% increase in the total clearance of *S*- and *R*-warfarin, was predicted with the coadministration of 100 mg CoQ_10_ [188]. CoQ_10_ may decrease the effects of warfarin [189], but results were inconsistent in another controlled, clinical trial [190]. In 171 patients, coadministration of CoQ_10_ with warfarin appeared to increase the risk of bleeding [191]. Clinical pharmacokinetic study suggested that CoQ_10_ had a prolonged elimination half-life (38–92 h) after oral administration [192].

#### 3.3.2. Glucosamine and Chondroitin Sulfate

Osteoarthritis (OA), a chronic illness and the most common of all the joint diseases, affects about 15% of the world’s population and is three times more common in women than in men [193]. Standard therapies can alleviate the symptoms of OA to some extent but cannot prevent disease progression. A number of alternative substances have been touted as beneficial for OA. Although their mode of action may be complex, glucosamine and chondroitin sulfate have been widely accepted as supplements in the management of OA because they are the essential components of proteoglycan in normal cartilage [194]. When a large-scale trial evaluated glucosamine and chondroitin sulfate alone or in combination, pain was not reduced in a group of patients with OA of the knee. Exploratory analyses suggested that the two in combination might be effective in a subgroup of patients with moderate-to-severe knee pain [195]. 

Long-term clinical data regarding the safety of glucosamine and chondroitin sulfate alone, or in combination, are limited. The use of chondroitin sulfate alone is well tolerated and without any significant adverse drug interaction [194]. One concern regarding the use of glucosamine is its potential to cause or worsen diabetes in animal models [196], an effect supported by clinical studies [197]. In a published report from the FDA MedWatch database, there were 20 reports of glucosamine or glucosamine-chondroitin sulfate use with warfarin. Coagulation was altered as manifested by increased INR or increased bleeding or bruising [198].

When glucosamine is taken orally, 90% of it is absorbed. Because of extensive first-pass metabolism, only 25% bioavailability is achieved by oral administration compared with a bioactivity of 96% with intravenous administration [199]. Peak plasma levels occurred 4 h after an oral dose and declined to baseline after about 48 h [200]. Chondroitin sulfate was absorbed slowly after oral ingestion with a plasma peak at 8.7 h and a decline to baseline at about 24 h [201]. 

#### 3.3.3. Fish Oil

The intake of fish oil supplements containing omega-3 fatty acids (EPA and DHA) reduces the incidence of many chronic diseases that involve inflammatory processes including cardiovascular diseases, inflammatory bowel disease, cancer, rheumatoid arthritis, and neurodegenerative illnesses [202]. In a recent study, however, omega-3 did not reduce the rate of death in patients with cardiovascular risk factors [203].

Omega-3 fatty acids, however, may inhibit platelet aggregation and increase the risk of bleeding. *In vitro* experiments have demonstrated an anti-platelet aggregatory effect of omega-3 fatty acids [204], and inhibition correlated with platelet cAMP levels [205]. *In vivo* studies show that omega-3 fatty acids decreased platelet aggregation but did not influence bleeding time [206,207]. In a clinical study, the inhibition of platelet aggregation by omega-3 fatty acid was gender-specific. EPA was significantly effective in reducing platelet aggregation in males, whereas DHA was not effective compared to the placebo. In females, DHA significantly reduced platelet aggregation but EPA did not [208]. 

Although evidence for significant bleeding concerns was not found in clinical trials [209,210], several case reports have illustrated a possible interaction between warfarin and omega-3 fatty acids [211]. Extremely elevated INR associated with warfarin in combination with omega-3 fatty acids was found in two cases [212,213]. In another case, after a minor fall, a patient developed a subdural hematoma requiring a craniotomy that likely was precipitated by concomitant use of high-dose omega-3 fatty acids with both aspirin and warfarin [214]. 

#### 3.3.4. Vitamins

Vitamins are organic compounds needed for normal metabolic functions. They are traditionally categorized as fat-soluble (vitamins A, D, E, and K) or water-soluble (vitamins B and C and biotin). Only vitamins C and E will be discussed here because of their potential effects related to coagulation and platelet function.

Vitamin C is a micronutrient essential for human health. Because humans cannot synthesize it, human survival depends on obtaining vitamin C from foods. The recommended dietary allowance (RDA) of vitamin C is 60 mg daily. The tolerable upper intake level is 2000 mg/day according to the Institute of Medicine [215]. The toxic effects of vitamin C are few and dose-dependent. Doses of vitamin C greater than 2000 mg/day have been associated with multiple adverse effects. Ingestion of 3 to 5 g at once may cause diarrhea and bloating [53]. Large doses may also precipitate hemolysis in patients with glucose 6-phosphate dehydrogenase deficiency [216]. One case report suggested that vitamin C might interfere with the action of warfarin [217], but other clinical studies did not support the theory [218,219]. 

Vitamin E is a potent lipid-soluble antioxidant in human plasma and tissues. It has many forms and biological activities. The most active form is α-tocopherol. The Food and Nutrition Board defined the human requirements for vitamin E as only α-tocopherol, specifically those forms with 2*R*-α-tocopherol stereochemistry [220]. The RDA for vitamin E is 15 mg/day (or 22.4 IU) [53], which can be met by dietary sources. The essential upper limit for safety is 1000 mg/day, but some experts advise avoiding supplementation with doses higher than 400 mg/day [221]. Vitamin E intakes are associated with an increased tendency to bleed and *in vitro* and *in vivo* studies showed that vitamin E inhibits platelet aggregation. A vitamin E quinone metabolite may be responsible for the effects of vitamin E [222], and the reduced platelet adhesiveness of vitamin E probably accompanies incorporation by plasma lipoproteins [223]. Risk of bleeding increased when vitamin E and warfarin were combined in an *in vitro* study [224]; however, this interaction was not supported by a preliminary clinical study [225]. Moderate doses of vitamin E (400 IU daily) increased the risk of bleeding because of inhibition of platelet aggregation and antagonism of vitamin K-dependent clotting factors [226]. In one clinical study, platelet adhesion decreased significantly in volunteers who took 400 IU of vitamin E plus 325 mg/day aspirin, compared to volunteers who took 325 mg/day of aspirin alone [227]. Data from another trial were unable to reproduce these results [228]. 

Other top ten dietary supplements include flaxseed oil, fiber or psyllium, cranberry, melatonin, methysulfonylmethane (MSM) and lutein [33]. No special concerns have been published associated with bleeding risk or other platelet function changes from the use of these supplements. 

## 4. Discussion

Although there are no official standards or guidelines on the preoperative use of alternative medicine, public and professional educational information advises that natural products be discontinued at least 2–3 weeks before surgery [38,229]. However, in practice, evaluating patients 2–3 weeks before elective surgery may not be practical. Moreover, some patients require non-elective surgery or are non-compliant with instructions to discontinue herbal medications preoperatively. These factors and the extensive use of herbal medicines may mean that herbal medications are taken until the time of surgery. Pharmacokinetic data on selected active constituents indicate that some herbal medications are eliminated quickly and may be discontinued close to the time of surgery.

The administration of warfarin in the perioperative period may be complicated by the potential drug–supplement interactions described above. Because the effects of dietary supplements on coagulation and platelet function are not well characterized and their effects are difficult to predict, it is prudent to advise their discontinuation before surgery [230].

Data on the safety of dietary supplements in the perioperative period are limited. Since patients often self-medicate with supplements without disclosure to their health care providers, physicians should ask patients about their present or past use of dietary supplements. A limitation of this review is that currently available data are mostly based on *in vivo* and limited clinical studies or case reports, whereas controlled clinical trials for natural products on coagulation and platelet function are largely unavailable. Nevertheless, we summarized potential perioperative effects of commonly used natural products. Data in this article can help physicians avoid potential problems associated with the use of natural products during the perioperative period.

## 5. Conclusions

Natural products are gaining popularity in the United States. There is extensive literature on the beneficial and adverse effects of natural products; however, safety data related to their bleeding risks are limited. To understand these potential complications of natural products, clinicians should explicitly elicit and document a history of natural product use by patients. In this article, we reviewed possible effects of natural products on coagulation and platelet function. Pharmacodynamic and pharmacokinetic information of commonly used herbal medicine and dietary supplements, and their interactions with anticoagulant medications were discussed. It is important for physicians and health care professionals to stay informed about commonly used natural products and their potential adverse effects, including those on coagulation and platelet function. 

## Abbreviations

CAM: Complementary and Alternative Medicine
OA: Osteoarthritis
EPA: Eicosapentaenoic Acid
DHA: Docosahexaenoic Acid
RDA: Recommended Dietary Allowance
MSM: Methysulfonylmethane
